# Prototypical Clinical Trial Registry Based on Fast Healthcare Interoperability Resources (FHIR): Design and Implementation Study

**DOI:** 10.2196/20470

**Published:** 2021-01-12

**Authors:** Christian Gulden, Romina Blasini, Azadeh Nassirian, Alexandra Stein, Fatma Betül Altun, Melanie Kirchner, Hans-Ulrich Prokosch, Martin Boeker

**Affiliations:** 1 Chair of Medical Informatics Department of Medical Informatics, Biometrics and Epidemiology Friedrich-Alexander University Erlangen-Nürnberg Erlangen Germany; 2 Institute of Medical Informatics Justus-Liebig-University Gießen Gießen Germany; 3 Carl Gustav Carus Faculty of Medicine, Center for Medical Informatics Institute for Medical Informatics and Biometry Dresden University of Technology Dresden Germany; 4 Institute for Community Medicine Section Epidemiology of Health Care and Community Health University Medicine Greifswald Greifswald Germany; 5 Medical Informatics Group University Hospital Frankfurt Frankfurt Germany; 6 Medical Center for Information and Communication Technology University Hospital Erlangen Erlangen Germany; 7 Institute of Medical Biometry and Statistics Medical Faculty and Medical Center University of Freiburg Freiburg Germany

**Keywords:** clinical trials, trials registry, health information interoperability, data sharing, HL7 FHIR

## Abstract

**Background:**

Clinical trial registries increase transparency in medical research by making information and results of planned, ongoing, and completed studies publicly available. However, the registration of clinical trials remains a time-consuming manual task complicated by the fact that the same studies often need to be registered in different registries with different data entry requirements and interfaces.

**Objective:**

This study investigates how Health Level 7 (HL7) Fast Healthcare Interoperability Resources (FHIR) may be used as a standardized format for exchanging and storing clinical trial records.

**Methods:**

We designed and prototypically implemented an open-source central trial registry containing records from university hospitals, which are automatically exported and updated by local study management systems.

**Results:**

We provided an architecture and implementation of a multisite clinical trials registry based on HL7 FHIR as a data storage and exchange format.

**Conclusions:**

The results show that FHIR resources establish a harmonized view of study information from heterogeneous sources by enabling automated data exchange between trial centers and central study registries.

## Introduction

Clinical trial registries establish publicly accessible databases about ongoing and completed clinical trials, aiding physicians and patients in selecting studies that are suitable for participation [1]. They help researchers identify related trials and are considered an essential tool for conducting systematic reviews [2]. Further, they increase the transparency and accountability of clinical research by identifying discrepancies between the original study design and results published in the literature [3,4]. Therefore, registration and maintenance of trial records can benefit patients and advance medical knowledge as a whole [5].

One challenge for researchers is keeping information up-to-date, especially across multiple study registries, each with a distinct data scheme and audience. In a 2017 study, Jones et al [6] analyzed the recruitment status of 405 trials registered on ClinicalTrials.gov and found that 31% either had an incorrect recruitment status specified or had a delay of more than 1 year between the time the study was concluded and the time the registry recruitment status was updated. Stergiopoulos et al [7] compared trial records from a commercial clinical trial database (Informa Pharma Intelligence's Trialtrove) with ClinicalTrials.gov and identified inconsistencies for site and enrollment information between the two databases [7].

The completeness and timeliness of study information may be improved by providing standardized interfaces to automatically create and update registry entries. These interfaces should be invoked by local systems that manage site-specific study information, such as recruitment status and contact details [8]. Such local registries for the documentation of trial metadata already exist at several sites for accounting, contract management, and electronic health record (EHR)-integration reasons [9,10]. Data from these local registries could be automatically exported to public external registries to provide an up-to-date view of the studies. However, this requires standardized interfaces and data models to ensure interoperability between these heterogeneous registries. Health Level 7 (HL7) Fast Healthcare Interoperability Resources (FHIR) is one such standard for modeling and exchanging health care–related data [11]. Resources are the fundamental building blocks of FHIR. Each resource defines a concrete clinical concept, such as patients (using the Patient resource), diagnoses (using the Condition resource), or an assessment of an allergy or intolerance (the AllergyIntolerance resource). Resources are composed of well-defined fields and data types and can be serialized using idiomatic JavaScript Object Notation (JSON) or XML. FHIR additionally defines a representational state transfer (REST) application programming interface (API) with a set of operations for creating, reading, updating, and deleting (CRUD) resources from a FHIR-compliant server.

In this study, we designed and implemented a multisite clinical trial registry based on the HL7 FHIR standard, which automatically collects up-to-date information on studies conducted across 10 university hospitals in Germany.

## Methods

### Design Objectives

The primary goal of this study is to provide access to current information on clinical studies conducted at participating university hospitals to interested parties via a web application. The secondary objective is to achieve a high degree of automation and standard compliance by utilizing HL7 FHIR. The standard does not limit the exact mechanism of transferring FHIR resources; however, it does specify a REST API for interacting with FHIR servers. The proposed trial registry design should leverage this interface specification for ease of implementation and better interoperability. All trial information should be automatically exported and updated from the site-local registry software systems, which were established as part of our previous work [8].

**Figure 1:**
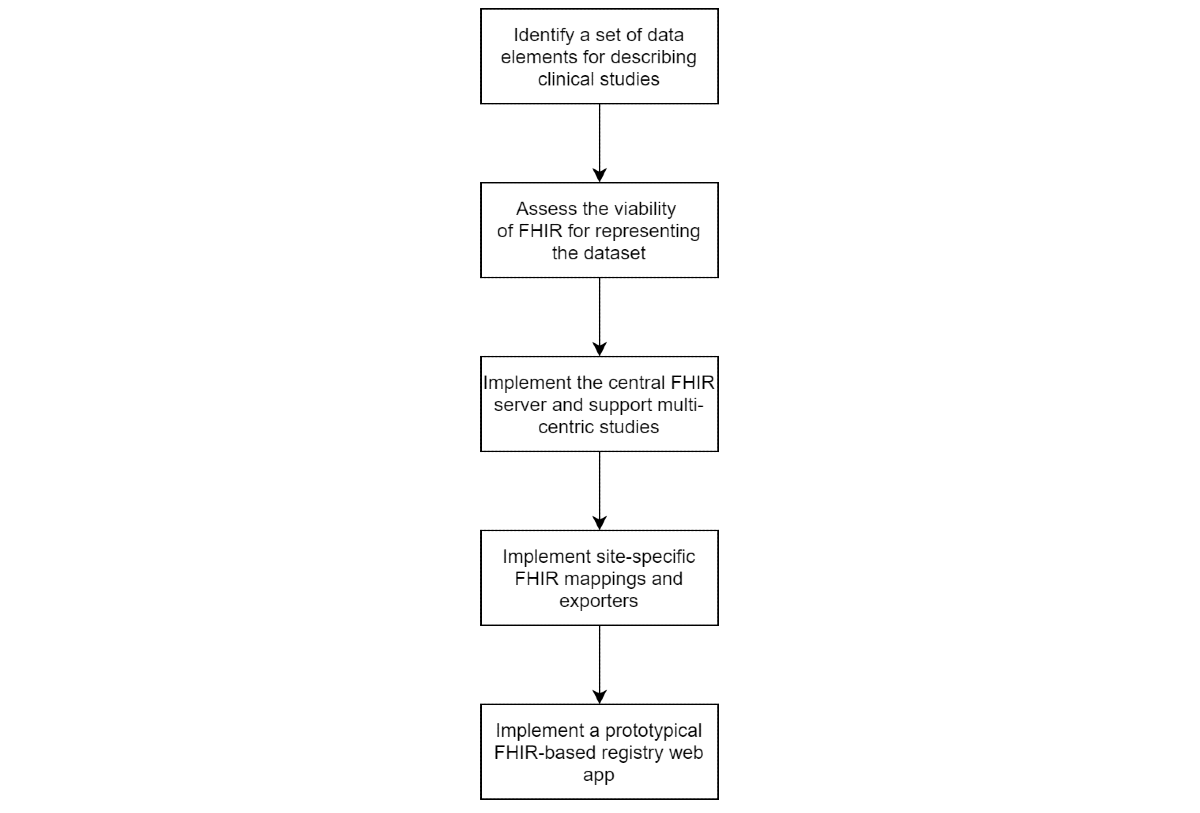
Flowchart showing the different phases of implementing the multisite Fast Healthcare Interoperability Resources (FHIR)-based trial registry.

The steps we have taken to implement the multisite clinical trial registry are outlined in Figure 1.

### Identifying a Set of Core Data Elements for Describing Clinical Studies

The data stored in the central trial registry is the basis for providing a website that allows physicians, researchers, and the public to search for and obtain information on clinical studies conducted at the participating sites. To determine what information should be included in the website, we analyzed the data structures used by the German clinical trials register (DRKS) [12], ClinicalTrials.gov [13], the World Health Organization (WHO) data set [14], and OpenTrials [15]. Additionally, we considered data elements already defined and used by the established local trial registries. For this purpose, we exported the data schemas and value ranges of these latter implementations. The 2 main criteria when deciding whether an individual data element should be included in the minimal data set were (1) its availability across all participating sites (ie, is the data element already documented in a structured way and accessible for export?), and (2) whether the data element is useful for a person seeking information on the study. As the data elements of the existing site-local registries were defined in collaboration with clinical stakeholders, they generally satisfied the second criteria. For example, Erlangen University Hospital initiated a working group in 2015 to define the requirements for a hospital-wide trials registry. Participants came from the hospital's center for clinical trials, the comprehensive cancer center, the major clinics pursuing clinical trials, and the hospital's IT department [9].

The different data schemas were compared and iteratively reduced until consensus was reached on a set of minimal data elements useful for providing basic information on running trials. This process was conducted collaboratively by one person from each of the 3 sites that had already implemented a trial registry. Therefore, the final data set was a tradeoff between data elements that were useful (criteria 2) and data elements that were available at all sites (criteria 1).

### Assessing the Viability of FHIR for Representing the Data Set

The HL7 FHIR standard defines a ResearchStudy resource representing information about a clinical study, such as its title, description, contact information, and recruitment status. Consequently, it can be used to exchange study protocol information [16]. We assessed whether this resource was suitable for representing all elements of the identified data set and whether extensions for application-specific profiles would need to be defined. If required, the profiles will be generated using the Forge tool (version 23.0; Firely) [17]. For this, an initial mapping between the data set and the elements of the FHIR ResearchStudy was proposed by one of the authors. Subsequently, this proposal was reviewed and commented on by the rest of the team in a collaborative way.

### Implementing Site-Specific FHIR Mappings and Exporters

In the next step, after identifying the required data elements, mappings were developed from the site-local study records to FHIR ResearchStudy resources. Additionally, functionality for transferring these resources to the central registry was implemented. The 10 sites participating in this study use a total of 5 distinct local study registries. A custom registry software, SODA, was initially developed by one site and was then co-developed and used by a total of 4 sites [8]. Here, the export functionality was implemented natively as a feature of the registry written in the Java programming language. Another 2 sites use the proprietary CentraXX Trial management software [17] and implemented a custom exporter using the Pentaho Data Integration ETL tool [18]. The remaining 4 sites use bespoke registry implementations, which made it necessary to write custom mappers and exporters implemented in Java and one in C#.

The mapping table created when assessing the viability of representing the data elements as FHIR ResearchStudy was used to guide the mapping process. Additionally, we used the local mappings created by one site as a reference to directly comment on and discuss the created resources.

## Results

### Core Data Set for Clinical Study Records and Its FHIR Mapping

**Figure 2:**
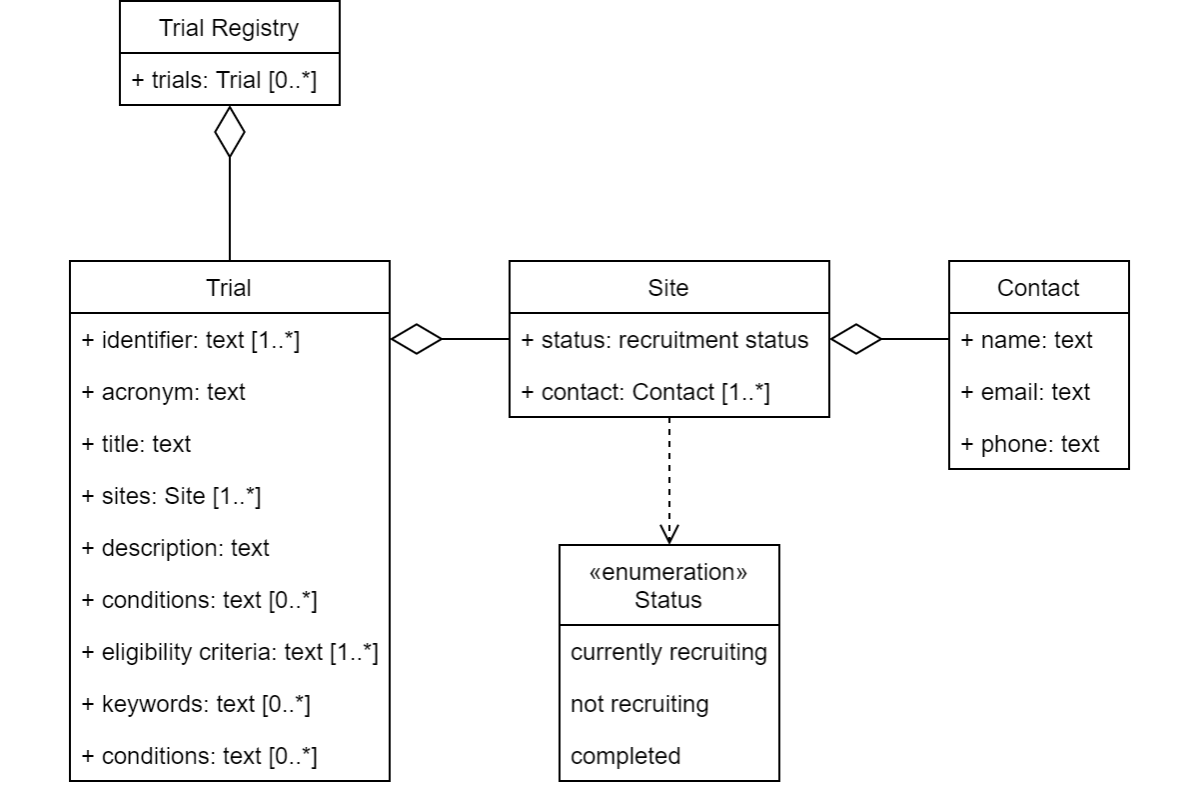
Unified Modeling Language (UML) diagram showing the set of identified data elements in the context of a trial registry.

We identified a set of 11 data elements that sufficiently communicate relevant study information to researchers, physicians, and patients (Table 1). The Unified Modeling Language (UML) diagram in Figure 2 shows how these elements fit into our high-level model of a trial registry: It manages an arbitrary number of trial objects, each with data fields containing relevant information about the trial. Because several investigational sites may participate in the same trial, there is a one-to-many relationship between the trial and site. In turn, each site can have several contact points for study inquiries.

Comparing our data elements with the definition of the FHIR ResearchStudy resource yielded an unambiguous mapping (Table 1). Table 1 also includes a column on the origin of the data element; if a direct equivalent in the WHO dataset exists, it is included in this column, as this dataset subsumes most other datasets (such as DRKS and ClinicalTrials.gov). If no direct equivalent could be found, the item in the ClinicalTrials.gov dataset is shown.

A limitation in the FHIR ResearchStudy specification is that the recruitment status can only be set per study and not per participating site. Similarly, while a list of contacts for study-related inquiries can be set on the resource (ResearchStudy.contact), these contacts are not explicitly linked to the study site to which they belong. Finally, the FHIR ResearchStudy, by default, does not allow for the specification of a study acronym. However, the FHIR standard allows for extending resources using custom profiles. This means that the available fields of the ResearchStudy resource can be extended in a structured way, and it can be verified whether a given instance adheres to the profile specification. We developed a FHIR profile which adds a per-site recruitment status, per-site contact information, and a field for the study acronym to the ResearchStudy. The profile is available online in the Simplifier repository [19].

The ResearchStudy.identifier field is used to specify site-local and global identifiers for a study. In FHIR, these identifiers are tuples consisting of a system (expressed as a URI) and a character string value. We created a table to map from common primary and secondary study numbers to these identifiers (Table 2). This table also includes mappings from identifying numbers to the corresponding web address in ResearchStudy.relatedArtifact.

**Table 1:** Mapping between the defined data elements (including their origins) and Fast Healthcare Interoperability Resources (FHIR) ResearchStudy resources. WHO: World Health Organization.

Core data set for study records	FHIR ResearchStudy	Origin
Identifier	ResearchStudy.identifier	WHO: Primary and Secondary Identifying Numbers
Acronym	*Custom Extension*	ClinicalTrials.gov: Acronym
Contact Details	ResearchStudy.contact	WHO: Contact for Public Queries, Contact for Scientific Queries
Participating Site	ResearchStudy.site	WHO: Countries of Recruitment; ClinicalTrials.gov: Location
Scientific Title	ResearchStudy.title	WHO: Public Title; Scientific Title
Description	ResearchStudy.description	ClinicalTrials.gov: Detailed Description
Conditions	ResearchStudy.condition	WHO: Health Condition(s) or Problem(s) Studied
Demographic Inclusion Criteria (gender and age)	ResearchStudy.enrollment	WHO: Key Inclusion and Exclusion Criteria
Recruitment Status	ResearchStudy.status	WHO: Recruitment Status
Further Information (URLs)	ResearchStudy.relatedArtifact	ClinicalTrials.gov: Link
Keywords	ResearchStudy.keyword	ClinicalTrials.gov: Keyword

**Table 2:** Mapping between various source registry identifying numbers and ResearchStudy.identifier systems and values. The mapping to ResearchStudy.relatedArtifact is also shown.

Identifier			RelatedArtifact		
Identifier Source	System	Example Value	URL	Label	Display
DRKS	http://www.drks.de	DRKS00000164	https://www.drks.de/drks_web/navigate.do?navigationId=trial.HTML&TRIAL_ID=DRKS00000164	DRKS00000164	DRKS
EudraCT	http://www.clinicaltrialsregister.eu	2012-000620-17	https://www.clinicaltrialsregister.eu/ctr-search/search?query=eudract_number:2012-000620-17	2012-000620-17	EudraCT
Universal Trial Number (UTN)	http://www.who.int/ictrp/unambiguous_identification/utn	U1111-1220-2928	*(no directly linkable URL available)*	U1111-1220-2928	UTN
ClinicalTrials.gov (NCT)	http://clinicaltrials.gov	NCT03521531	https://clinicaltrials.gov/ct2/show/NCT03521531	NCT03521531	ClinicalTrials.gov
site-specific/local Ids	*(Example)* https://fhir.uk-erlangen.de/studienregister/NamingSystem/id	rvnoqjmezlew	*(Example)* https://studienregister.uk-erlangen.de/details/rvnoqjmezlew	rvnoqjmezlew	Trials Registry University Hospital Erlangen

### Central Trial Registry

#### Architecture of the FHIR-based Trial Registry

**Figure 3:**
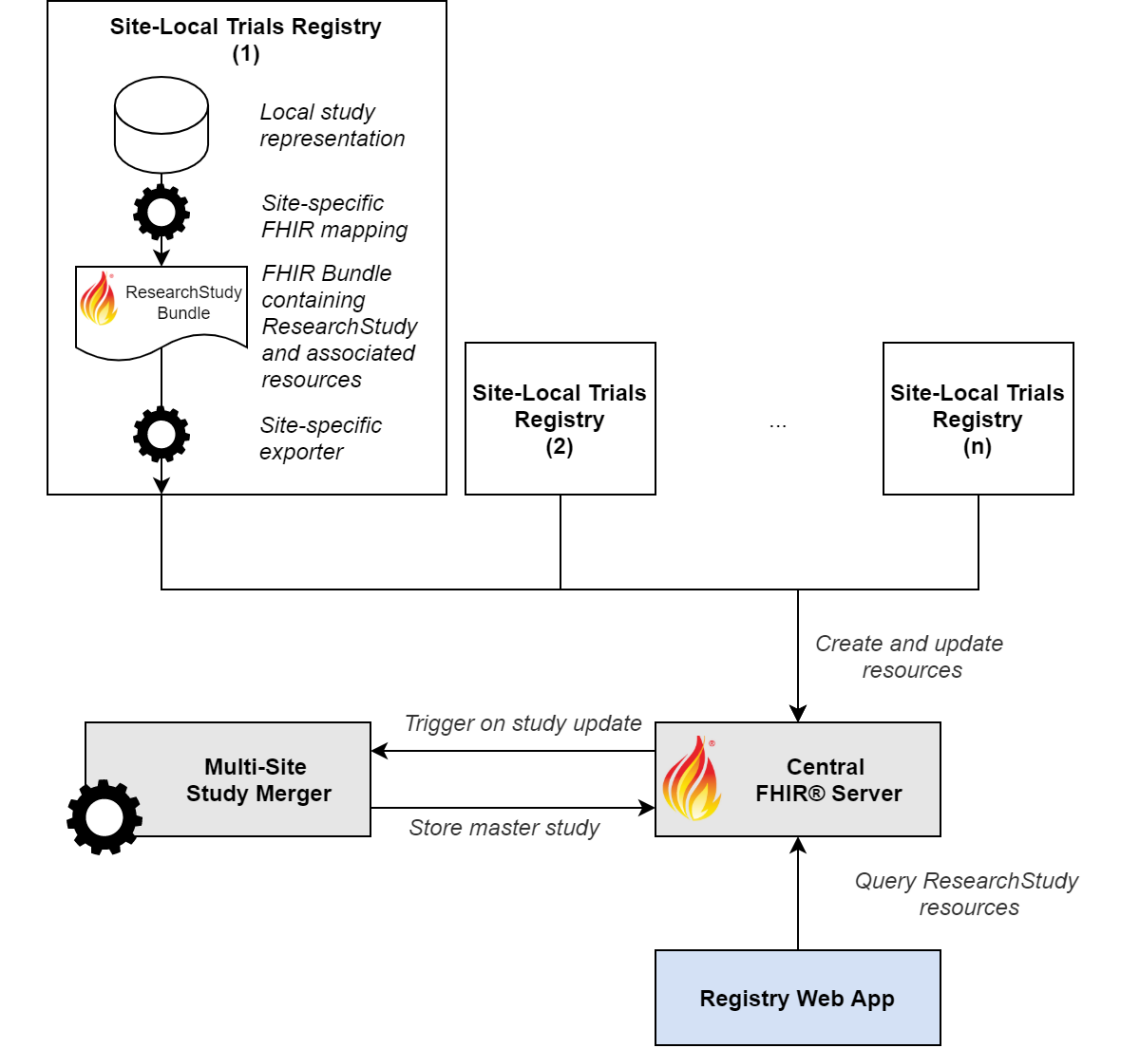
Architecture of the Fast Healthcare Interoperability Resource (FHIR)-based trial registry.

The architecture of the central registry is centered around a single standard-compliant FHIR server (Figure 3). The site-local registries continuously export and update the site-specific ResearchStudy records using the FHIR REST interface. The web application displaying the studies interacts with the FHIR server via the same API in a read-only fashion. All design decisions and implementations are based on FHIR Release 4.0.1 (HL7). The central trial registry is implemented based on a HAPI FHIR server (version 5.0.2; Smile CDR) [20] using a PostgreSQL database (version 12.3; PostgreSQL Global Development Group) for storage [21]. The central registry components were deployed on an on-premise Kubernetes cluster (version 1.18; Cloud Native Computing Foundation) [22].

#### Local Registry Mappers and Exporters

The implementation details of the exporters vary from site to site, depending on the software used. In general, logic was written to map the study representations from the local registries to FHIR ResearchStudy and any additional resources required. The latter consist of the FHIR Location resource to identify the site (referenced by ResearchStudy.site) and the FHIR Group resource (referenced by ResearchStudy.enrollment) used to define the eligibility criteria. The exports are generally implemented as a single FHIR transaction bundle containing all study records per site. Some implementations additionally allow for automatically exporting and updating individual study records whenever the data in the local registry changes. In either case, standard FHIR REST semantics are used when interacting with the central server. An example of a mapped clinical trial is included in Multimedia Appendix 1.

#### Merging Multicentric Studies

**Figure 4:**
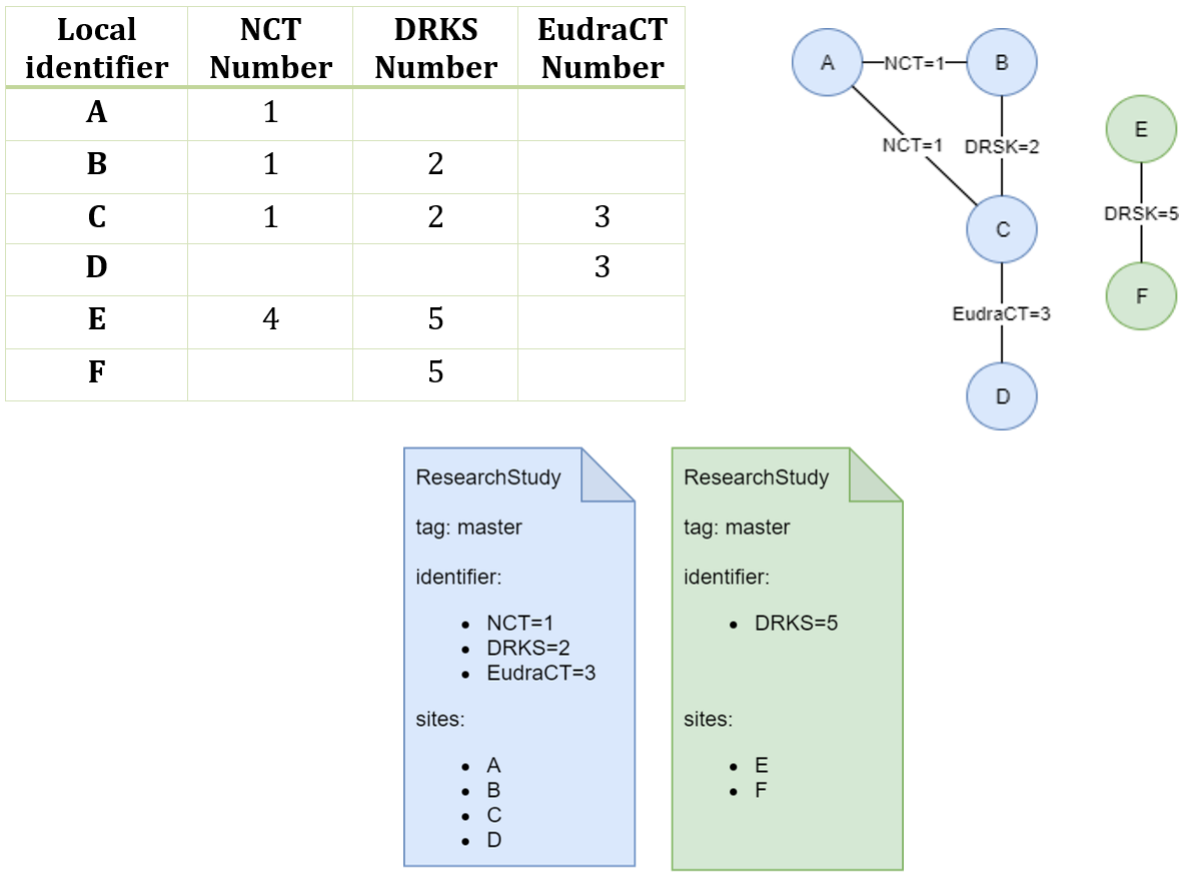
Example of 6 exported records, 4 of which (A-D) refer to one multicentric study (NCT=1, DRKS=2, EudraCT=3), and 2 of which (E and F) refer to a different multicentric study (NCT=4, DRKS=5), represented as a table (left) and an undirected graph (right). These studies are merged into 2 master ResearchStudy resources, each with a distinct set of identifiers and participating sites (bottom).

In our design, all site-local registries create and update their study records independently; however, in cases of a multicentric study with more than one participating site, this results in redundant ResearchStudy resources being stored in the FHIR server. To intercept such cases, the standard FHIR server is extended with a custom module (the multisite study merger), which creates a master record for each distinct study in the server. The registry's web interface only displays these master records. Multicentric studies are identified as ResearchStudy resources in the server that were exported by different sites (different local study registries) while having the same primary identifiers. We used the unique identifiers assigned by ClincalTrials.gov (NCT number), DRKS (DRKS number), and the European Union Drug Regulating Authorities Clinical Trials Database (EudraCT number) as primary identifiers. Due to data quality issues in the local source systems, not all of these primary identifiers may be set for all exported studies, although the actual studies are registered in one of the above registries. An example of such a case, and the problem arising from it, is shown in Figure 4. Here, all 4 of the local study records (A-D) refer to the same multicentric study with primary identifiers of 1 (NCT number), 2 (DRKS), and 3 (EudraCT), while another set of 2 local records refer to the same study identified by NCT number 4 and DRKS number 5. The challenge lies in identifying that A-D and E-F represent 2 distinct studies. In the visualization of the records as a graph, each vertex is a local study resource and each edge represents a shared primary identifier (Figure 4). Creating such a graph from all records in the FHIR server reduces the identification of multicentric studies to extracting all connected components from it. The multisite study merger implements this by first retrieving all ResearchStudy resources from the central FHIR server. Next, an undirected graph is constructed where each ResearchStudy is stored as a vertex, and its list of identifiers are added as edges connected to all other ResearchStudy nodes with the same identifier. To find all the connected components in this graph, a breadth-first search is conducted, starting from each unvisited vertex in the graph and recursively visiting all neighbors until none remain. The algorithm is implemented using the JGraphT library [23]. Each connected component—that is, each list of ResearchStudy resources with the same common identifiers—is now merged into a single master study. This master study contains a list of distinct identifiers, keywords, and conditions of all studies in the set. The contact details and recruitment status are converted into extensions on the record in accordance with the FHIR profile defined in section “Core Data Set for Clinical Study Records and Its FHIR Mapping.” These studies are marked using a “master” tag in the FHIR ResearchStudy metadata field. Each master record is thereby uniquely identified by the presence of the master tag and any of its identifiers. A transaction implemented as a conditional update containing the master records is finally sent to the FHIR server. The implementation can handle both the addition and removal of local study resources and updates the master records accordingly. The source code of this application is available online [24].

#### Registry Web Application

**Figure 5:**
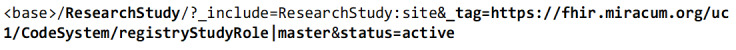
The HTTP GET query sent to the Fast Healthcare Interoperability Resources (FHIR) server to retrieve all actively recruiting master studies.

The web frontend for the trial registry is implemented as a single-page SMART-on-FHIR [25] VueJS application. It uses the REST API of the central FHIR server to retrieve all master study records. The query to the server is shown in Figure 5. It requests all FHIR ResearchStudy resources that are actively recruiting (status=active) and that are tagged “master” studies (&_tag=https://fhir.miracum.org/uc1/CodeSystem/registry StudyRole|master), a required filter, as otherwise, all site-specific studies are returned as well.

Once all studies are returned from the FHIR server, they are displayed to the user. The web interface allows for filtering studies by site and provides a basic free-text search functionality implemented using the client-side Fuse.JS JavaScript library [26]. At the time of writing, 2542 studies have been exported to the central registry. After merging, 2099 distinct master study records remain, of which 925 are actively recruiting and displayed on the website. The web app is accessible online [27], and the source code is available [24]. Screenshots of the app are displayed in Multimedia Appendix 2.

## Discussion

### Principal Findings

In this study, we investigated how a common, standard representation of clinical trials can be used to implement a central trials registry that receives and merges data from heterogenous study registries. We leveraged HL7 FHIR for this purpose.

With the design and development of an open-source central trial registry containing records from university hospitals, we provided an architecture and implementation of a multisite clinical trials registry based on HL7 FHIR as a data storage and exchange format. The results show that FHIR resources establish a harmonized view of study information from heterogeneous sources by enabling automated data exchange between trial centers and central study registries.

### Comparing FHIR to Alternative Representations

Similar to our attempts to harmonize data from heterogeneous trial registries, the Observational Medical Outcomes Partnership Common Data Model (OMOP CDM) is used to store and analyze observational health data from disparate source databases [28]. The OMOP CDM is a patient-centric data model containing clinical data that is mapped to a set of standard terminologies. By default, the schema does not provide a way to store study information. However, in July 2020, a proposal was created by the Observational Health Data Science Informatics (OHDSI) Clinical Trials Working Group to define conventions for storing trial metadata, patient enrollment, and other observationally relevant data with minimal extensions to the schema [29]. The focus of this effort is to model the relationship between patients and clinical trials for research. This means that the suggested data elements are not as suitable for completely representing the meta-information of clinical trials as those available in a FHIR ResearchStudy.

In OMOP CDM, the extensive use of standardized terminologies (such as LOINC, ICD, and SNOMED CT) makes it possible to share queries and analytical applications between databases conforming to the CDM. Similarly, FHIR ensures interoperability between systems by including a reference to a terminology or ontology when specifying a code or value. FHIR profiles can be used to enforce the terminologies to use. For example, the ResearchStudy profile we defined requires that the “Health Condition(s) or Problem(s) Studied” characteristic of a study (ResearchStudy.condition) be provided as either ICD-10-GM (International Statistical Classification of Disease and Related Health Problems, 10^th^ revision, German Modification) or SnomedCT codes.

CDISC’s (Clinical Data Interchange Standards Consortium) Clinical Trial Registry (CTR)-XML, version 1.0, is standard based on a single XML file that can be used to generate submissions to the WHO, European Medicines Agency (EMA), EudraCT, and ClinicalTrials.gov registry [30]. CDISC also defines the Protocol Representation Model (PRM), a conceptual model for organizing a study protocol [31]. However, we were unable to find concrete implementations of either standard demonstrating the exchange of study information with a registry. In comparison, FHIR's open ecosystem and strong industry adoption provided tooling and libraries in several programming languages, helping us rapidly implement the site-specific mappings, exporters, and components of the central registry. Additionally, the specification of the RESTful web services in FHIR made providing a central server with a well-specified interface trivial. Support for RESTful web services has been recommended as a future research direction for the CDISC ODM by Hume et al [32].

Although FHIR promises semantic interoperability, in practice, we still encountered issues that required communication and manual review to resolve: technical problems like text encoding, trailing whitespaces in identifiers causing the merging process to run incorrectly, or timeouts in the central FHIR server when the received transactions contained too many resources.

### Extensions to the ResearchStudy Resource

We defined a custom profile on the default FHIR ResearchStudy resource to represent the study acronym, the recruitment status, and contact details for each participating site. Additional extensions are expected to be necessary when representing study details beyond our minimal study record data set. As such, subjects for future work should include analyses of how well the complete data structures used in existing trial registries can be mapped to the FHIR ResearchStudy resource and whether additional profiles or modifications to the base resource are necessary. In particular, previous studies on the usability of existing clinical trial registries have found that the inclusion of a lay summary has a substantial effect on the accessibility of clinical trial information for the general public [33,34]. At the time of writing, the FHIR ResearchStudy resource is at the “Trial Use” level of maturity, thus allowing our findings to influence the future development of the resource.

### Representation of Eligibility Criteria

Clinical trial eligibility criteria are usually expressed in human-readable text, which is challenging to process computationally [35,36]. In the FHIR ResearchStudy, eligibility criteria can be specified in the enrollment field, which does not dictate the exact format of the criteria. In our implementation, we represented the demographic criteria gender and age as a simple code and value range datatype, respectively. More complex eligibility criteria can be stored in arbitrary textual or binary representations and referenced by the study resource. This is useful because, while no single, standard computable format for clinical trial eligibility criteria exists [37], the FHIR ResearchStudy provides a framework for semantically annotating and exchanging recruitment logic in a standardized manner. For example, the OHDSI ATLAS tool can be used to create patient cohorts from an OMOP CDM database [38]. This is an important feature, especially if the trial registry is used as part of a larger system to support the patient recruitment process [39].

### Handling Inconsistent Data

When merging multiple studies into a single master study record, shared attributes, such as the title, description, or acronym, are arbitrarily taken from the first study where these values are available. However, there are cases where these shared attributes differ between multiple studies. To give a concrete example, the clinical trial with NCT number NCT02393859 has a total of 5 different known titles: the brief and official title used by ClinicalTrials.gov, the full and layperson title from EudraCT, and the title from the study protocol document. One site uses the official title from ClinicalTrials.gov, as the study was originally imported from there into the local system, whereas another site uses the title from the protocol document. A comparison of these titles is shown in Table 3 (note the addition of the word “Adaptive” in the title from the study protocol document). In this case, there is also an additional difference in the casing of the word “with;” however, the similarity comparison used in the merging algorithm is case-invariant.

**Table 3:** Comparison of different study titles for the NCT02393859 trial. Titles were copied verbatim from [40] and [41].

Title	ClinicalTrials.gov	EudraCT	Study protocol document
Official title/full title	Phase 3 Trial to Investigate the Efficacy, Safety, and Tolerability of Blinatumomab as Consolidation Therapy Versus Conventional Consolidation Chemotherapy in Pediatric Subjects With HR First Relapse B-precursor ALL	A Randomized, Open-label, Controlled Phase 3 Trial to Investigate the Efficacy, Safety, and Tolerability of the BiTE® Antibody Blinatumomab as Consolidation Therapy Versus Conventional Consolidation Chemotherapy in Pediatric Subjects with High-risk First Relapse B-precursor Acute Lymphoblastic Leukemia (ALL)	A Randomized, Open-label, Controlled Phase 3 Adaptive Trial to Investigate the Efficacy, Safety, and Tolerability of the BiTE® Antibody Blinatumomab as Consolidation Therapy Versus Conventional Consolidation Chemotherapy in Pediatric Subjects With High-risk First Relapse B-precursor Acute Lymphoblastic Leukemia (ALL)
Brief title/lay title	Phase 3 Trial of Blinatumomab vs Standard Chemotherapy in Pediatric Subjects With HIgh-Risk (HR) First Relapse B-precursor Acute Lymphoblastic Leukemia (ALL)	Clinical Study to Investigate the Efficacy, Safety, and Tolerability of the bispecific antibody Blinatumomab as Consolidation Therapy Versus Conventional Consolidation Chemotherapy in Pediatric Subjects with High-risk First Relapse Acute Lymphoblastic Leukemia (ALL)	N/A^1^

^1^ N/A: not applicable.

The differences between these titles may result from the initial study entry into the different primary registries, but it is also possible that amendments may have changed them. Given the asynchronous and distributed nature of our implementation, some sites might be exporting the updated study description while others are not. For the central multisite merging process, it is impossible to automatically determine which trial title is the correct one without additional input.

We currently log such cases and attempt to resolve them manually by communicating the discrepancies between the sites. These issues could be avoided if a “single source of truth” record was defined whose values are used in case of discrepancy.

To quantify this issue, we analyzed the number of multisite trials where intersite differences between the data elements study title, description, and acronym were present. We used the list of study clusters (ie, the list of connected components) in which each element represents one site-local ResearchStudy that belongs to the same multisite study, and determined the number of unique values for each data element within the same cluster. If this number was larger than one for a cluster and a data element, it indicated that there is a difference between at least 2 of the sites. We ignored cases where one of the values was not set, as this does not indicate a conflict that would need to be resolved. Before comparing the text values, all whitespaces were normalized to a single space, and all text was lowercased. This ensures that details that would only affect the display did not affect the results. Of the total 2542 exported studies, 769 were multicentric studies with at least 2 participating sites. Table 4 shows the results of this analysis.

**Table 4:** Multisite studies in which a difference in value was present between at least 2 site-local study records. For example, in 34 of the 769 multisite studies, there were 2 or more different values for the German study title.

Study record feature	Multisite studies in which a difference in value exists between at least 2 sites, n (%)
Acronym	96 (12.5)
Title (German)	34 (4.42)
Title (English)	105 (13.7)
Description (German)	5 (0.65)
Description (English)	51(6.63)

### Alternative Implementations Considered

Before settling on implementing a centralized FHIR-server–based architecture, we considered a federated approach: instead of local registries mapping and exporting their studies to a central server, each site would implement a FHIR REST façade on top of their local study registries. The website component would then query, aggregate, and display studies from all sites on each request. This approach is challenging as it requires both low latency and high availability of all sites. Besides these concerns regarding scalability and robustness, security concerns were raised, given that this would require external access to the hospital's network.

Instead of storing all studies exported by all sites and the master study records, it would be sufficient to just store the master record of each distinct study and have the local registries update the recruitment status or contact details for their site. This can be implemented using REST's PATCH semantics. However, in practice, this has the main disadvantage of increasing the complexity of the clients, as special care must be taken to avoid issues when concurrently writing to the same resource. Further, storing the complete study records per site in the FHIR server has advantages: It allows us to track changes to the resources over time, and analyze discrepancies in the completeness and quality of the study metadata between sites by using the FHIR history feature, which provides an audit trail for each change [42].

### Limitations and Future Work

As an initial, technical proof-of-concept, the registry presented in this study has several limitations and opportunities for future improvements.

The current implementation of the multisite merging algorithm requires all studies to be retrieved from the FHIR server before being merged, and the master studies to be updated. At our current scale of a few thousand studies, and because we are currently only running the merging process once a day, the overhead of processing more than just the changed studies was tolerable. However, instead, a more scalable implementation should identify and process only those resources that are affected by an update to a given ResearchStudy resource. This may be achieved by recursively retrieving all ResearchStudy resources with the same identifiers as the updated study or by persisting and updating the studies' graph representation.

The study was conducted within a small consortium, making it easy to manually review and give feedback on the study exports of the participating sites to resolve data quality and mapping issues. This manual approach for handling data discrepancies will need to be revised to support the use at scale.

We only provided a very basic implementation of a web interface. A thorough usability and requirements analysis from an end-user point of view may reveal additional information that should be included as part of the ResearchStudy resources. While the usefulness of the data elements we selected for display was assessed by clinical experts, and these elements largely overlap with the WHO data set, a formal evaluation of their adequateness, especially from the perspective of the general public, is still required. However, a recent online-survey to determine patient preferences when searching for clinical trials for participation concluded that “when searching for clinical trials, survey participants rated condition (66.4%), trial location (57.0%), trial dates (52.9%), age and gender (48.6%), and health measurements (ie, what the study measures; 45.5%) as the most important items” [43], items that are already represented in the resource and identified as part of our core data set.

Within the Medical Informatics for Research and Care in University Medicine (MIRACUM) consortium, we are currently implementing a clinical trial recruitment support system based on FHIR and the OMOP CDM [39]. The system will propose potential candidates for selected clinical trials based on data available in the EHR. In an initial version, the central trials registry described in this study will be used to provide FHIR ResearchStudy resources, which can be referenced by the FHIR ResearchSubject resources used to represent potential candidates. In later iterations, we plan on using the central registry to exchange computable trial eligibility criteria. This will allow us to create trial recommendations for trials that may be conducted at any of the participating sites.

### Conclusions

The scientific community and the public have a great need for standardized study registration to increase transparency in medical research by making information and results of planned, ongoing, and completed studies publicly available. The WHO Trial Registration Data Set specifies 24 data items that should be defined for a study in order to be considered fully registered; however, it does not define a structured exchange format for these items, leading to duplicate entries of study information and a lack of interoperability between trial registries. In this study, we have shown how HL7 FHIR can fill this role by developing a prototypical implementation. Additional work is necessary to refine the functionality and evaluate whether it can realistically reduce manual documentation and registration efforts at scale. We recommend that maintainers of trial registries investigate supporting FHIR as a standardized format based on our findings.

## Appendix

Multimedia Appendix 1 Sample Fast Healthcare Interoperability Resources (FHIR) ResearchStudy resource in JSON format, containing a master study record with 2 participating sites.

Multimedia Appendix 2 Screenshots of the trials registry web app.

## Abbreviations

API: application programming interface
CDISC: Clinical Data Interchange Standards Consortium
CDM: common data model
EHR: electronic health record
FHIR: Fast Healthcare Interoperability Resources
HL7: Health Level 7
MIRACUM: Medical Informatics for Research and Care in University Medicine
OMOP CDM: Observational Medical Outcomes Partnership Common Data Model
REST: representational state transfer
WHO: World Health Organization
