# Supplementary material for: Prototypical Clinical Trial Registry Based on Fast Healthcare Interoperability Resources (FHIR): Design and Implementation Study
Source: JMIR Med Inform. 2021 Jan 12;9(1):e20470. doi: 10.2196/20470 (PMC7837997; doi:10.2196/20470)
Supplement: Multimedia Appendix 1 [file medinform_v9i1e20470_app1.docx]

Multimedia Appendix

# Appendix 1

## Sample FHIR ResearchStudy resource in JSON format containing a master study record with two participating sites:

{

  "resourceType": "ResearchStudy",

  "id": "16588",

  "meta": {

    "versionId": "4",

    "lastUpdated": "2020-05-18T01:05:12.069+00:00",

    "source": "#AnXF9ztCqz78qfFm",

    "tag": [

      {

        "system": "https://fhir.miracum.org/uc1/CodeSystem/registryStudyRole",

        "code": "master"

      }

    ]

  },

  "contained": [

    {

      "resourceType": "Group",

      "id": "1",

      "active": true,

      "type": "person",

      "actual": false,

      "name": "Eligibility criteria for the 'BYLIEVE' study.",

      "characteristic": [

        {

          "code": {

            "coding": [

              {

                "system": "http://terminology.hl7.org/CodeSystem/usage-context-type",

                "code": "age"

              }

            ]

          },

          "valueRange": {

            "low": {

              "value": 18,

              "system": "http://hl7.org/fhir/ValueSet/age-units",

              "code": "a"

            }

          },

          "exclude": false

        }

      ]

    }

  ],

  "extension": [

    {

      "url": "https://fhir.miracum.org/uc1/StructureDefinition/studyAcronym",

      "valueString": "BYLIEVE"

    }

  ],

  "identifier": [

    {

      "use": "official",

      "system": "http://www.clinicaltrialsregister.eu",

      "value": "2016-004586-67"

    },

    {

      "use": "usual",

      "system": "http://unklinikum-dresden.de/SponsorCode",

      "value": "D-V"

    },

    {

      "use": "secondary",

      "system": "https://fhir.uk-erlangen.de/NamingSystem/identifier/studyId",

      "value": "epjklerybqnr"

    },

    {

      "use": "usual",

      "system": "https://www.uniklinikum-dresden.de/de",

      "value": "900-000001299"

    },

    {

      "use": "official",

      "system": "http://clinicaltrials.gov",

      "value": "NCT03056755"

    }

  ],

  "title": "BYLieve: A phase II, multicenter, open-label, three-cohort,non-comparative study to assess the efficacy and safety of alpelisib plus fulvestrant or letrozole in patients with PIK3CA mutant, hormone receptor (HR) positive, HER2-negative advanced breast cancer (aBC), who have progressed on or after prior treatments",

  "status": "active",

  "condition": [

    {

      "coding": [

        {

          "system": "http://fhir.de/CodeSystem/dimdi/icd-10-gm",

          "code": "C50"

        }

      ],

      "text": "Bösartige Neubildung der Brustdrüse [Mamma]"

    }

  ],

  "relatedArtifact": [

    {

      "type": "documentation",

      "label": "2016-004586-67",

      "display": "EudraCT",

      "url": "https://www.clinicaltrialsregister.eu/ctr-search/search?query=2016-004586-67"

    },

    {

      "type": "documentation",

      "label": "NCT03056755",

      "display": "ClinicalTrials.gov",

      "url": "https://clinicaltrials.gov/ct2/show/study/NCT03056755"

    }

  ],

  "keyword": [

    {

      "coding": [

        {

          "system": "https://fhir.uk-erlangen.de/CodeSystem/studyCategory",

          "code": "entgyn"

        }

      ],

      "text": "Gynäkologische Tumore (z.B. Brustkrebs, Gebärmutterkrebs)"

    },

    {

      "coding": [

        {

          "system": "https://fhir.uk-erlangen.de/CodeSystem/studyCategory",

          "code": "gynmamma"

        }

      ],

      "text": "Brustkrebs (Mammakarzinom)"

    }

  ],

  "description": "Eine multizentrische, offene, nicht-vergleichende Phase II-Studie mit zwei Kohorten zur Untersuchung der Wirksamkeit und Sicherheit von Alpelisib plus Fulvestrant oder Letrozol bei Patienten mit PIK3CA-mutierten, Hormonrezeptor positiven (HR+), HER2 negativen (HER2-) fortgeschrittenen Brustkrebs (aBC), die unter oder nach einer Therapie mit einem CDK4/6-Inhibitor einen Progress erlitten haben.",

  "enrollment": [

    {

      "reference": "#1"

    }

  ],

  "site": [

    {

      "extension": [

        {

          "url": "https://fhir.miracum.org/uc1/StructureDefinition/siteRecruitmentStatus",

          "valueCodeableConcept": {

            "coding": [

              {

                "system": "http://hl7.org/fhir/research-study-status",

                "code": "active"

              }

            ]

          }

        },

        {

          "url": "https://fhir.miracum.org/uc1/StructureDefinition/siteContact",

          "valueContactDetail": {

            "name": "Studienzentrum Frauenklinik",

            "telecom": [

              {

                "system": "email",

                "value": "fk-studienzentrale@<REDACTED>",

                "use": "work",

                "rank": 1

              },

              {

                "system": "phone",

                "value": "+49 9131-<REDACTED>",

                "use": "work",

                "rank": 1

              }

            ]

          }

        }

      ],

      "reference": "Location/1"

    },

    {

      "extension": [

        {

          "url": "https://fhir.miracum.org/uc1/StructureDefinition/siteRecruitmentStatus",

          "valueCodeableConcept": {

            "coding": [

              {

                "system": "http://hl7.org/fhir/research-study-status",

                "code": "active"

              }

            ]

          }

        },

        {

          "url": "https://fhir.miracum.org/uc1/StructureDefinition/siteContact",

          "valueContactDetail": {

            "name": "C <REDACTED>",

            "telecom": [

              {

                "system": "email",

                "value": "studiensekretariat.gyn@<REDACTED>",

                "use": "work",

                "rank": 1

              },

              {

                "system": "phone",

                "value": "+49 351-<REDACTED>",

                "use": "work",

                "rank": 1

              }

            ]

          }

        },

        {

          "url": "https://fhir.miracum.org/uc1/StructureDefinition/siteContact",

          "valueContactDetail": {

            "name": "Dr. <REDACTED>",

            "telecom": [

              {

                "system": "email",

                "value": "<REDACTED>",

                "use": "work",

                "rank": 1

              }

            ]

          }

        },

        {

          "url": "https://fhir.miracum.org/uc1/StructureDefinition/siteContact",

          "valueContactDetail": {

            "name": "Prof. Dr. med. <REDACTED>",

            "telecom": [

              {

                "system": "email",

                "value": "<REDACTED>",

                "use": "work",

                "rank": 1

              }

            ]

          }

        }

      ],

      "reference": "Location/8892"

    }

  ]

}
