# Supplementary material for: Prototypical Clinical Trial Registry Based on Fast Healthcare Interoperability Resources (FHIR): Design and Implementation Study
Source: JMIR Med Inform. 2021 Jan 12;9(1):e20470. doi: 10.2196/20470 (PMC7837997; doi:10.2196/20470)

Multimedia Appendix

# Appendix 2

## Initial view of the registry website after loading


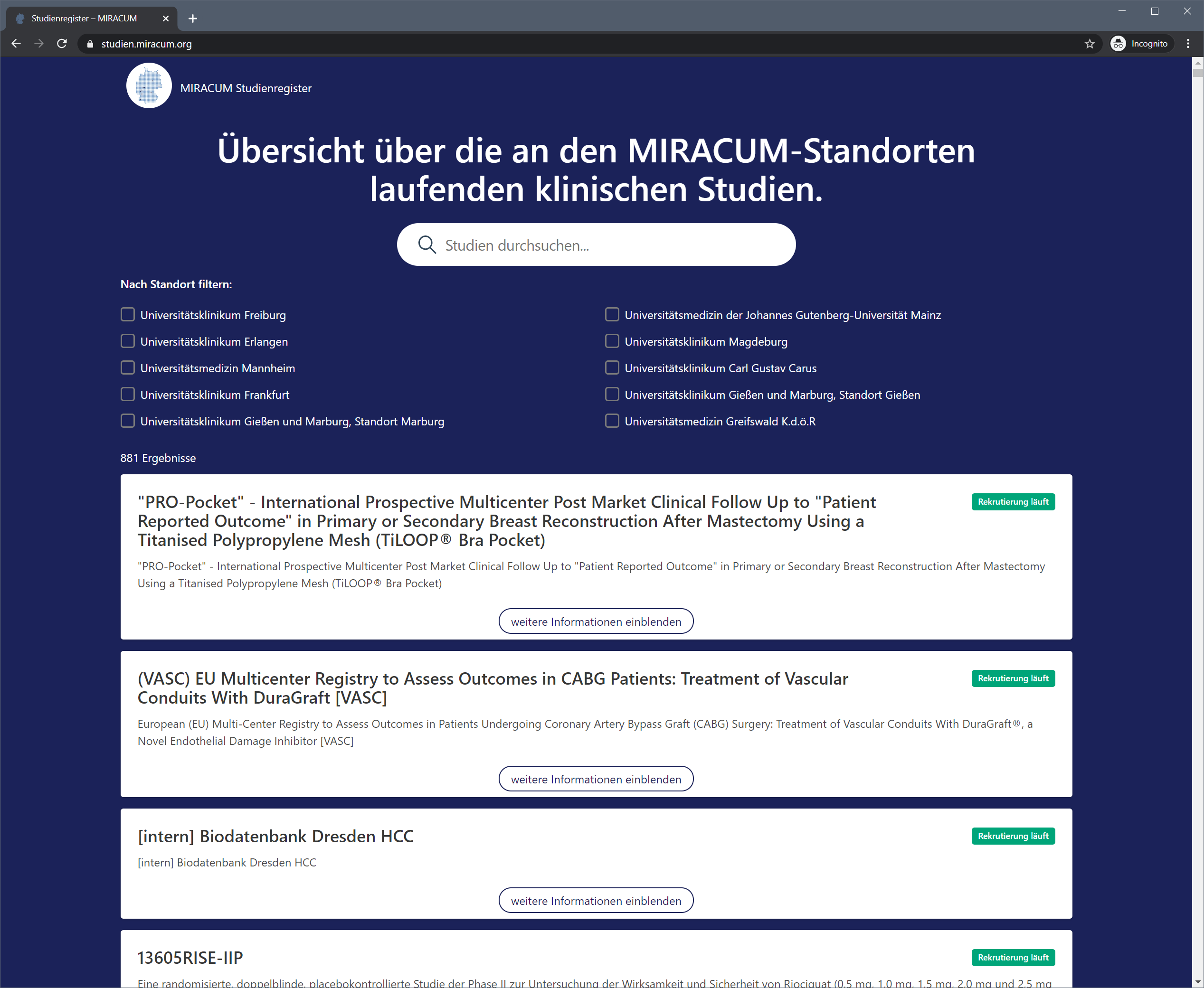


## Expanded view of a study record


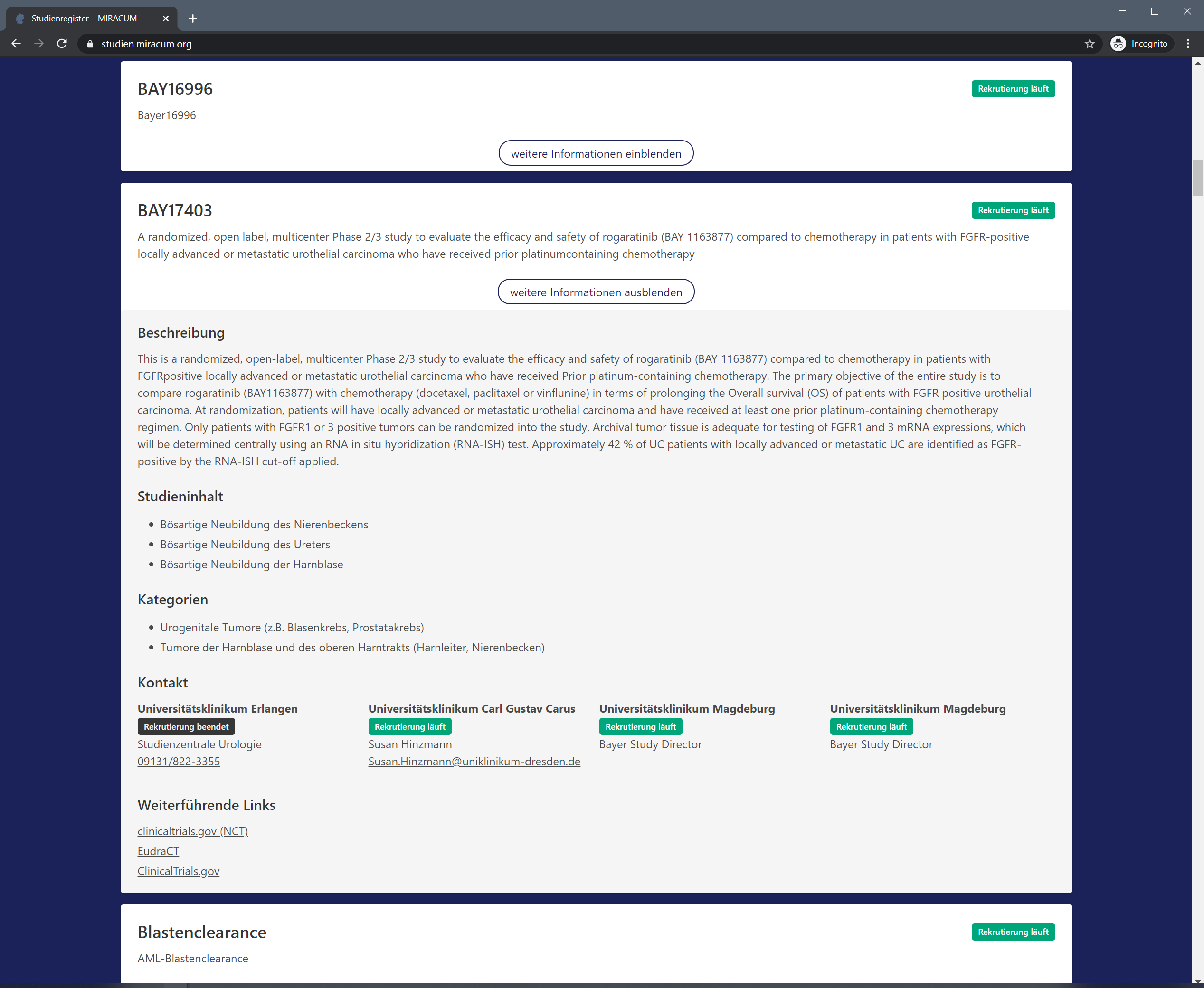


## Search Results after filtering by site and keyword “Leukemia”


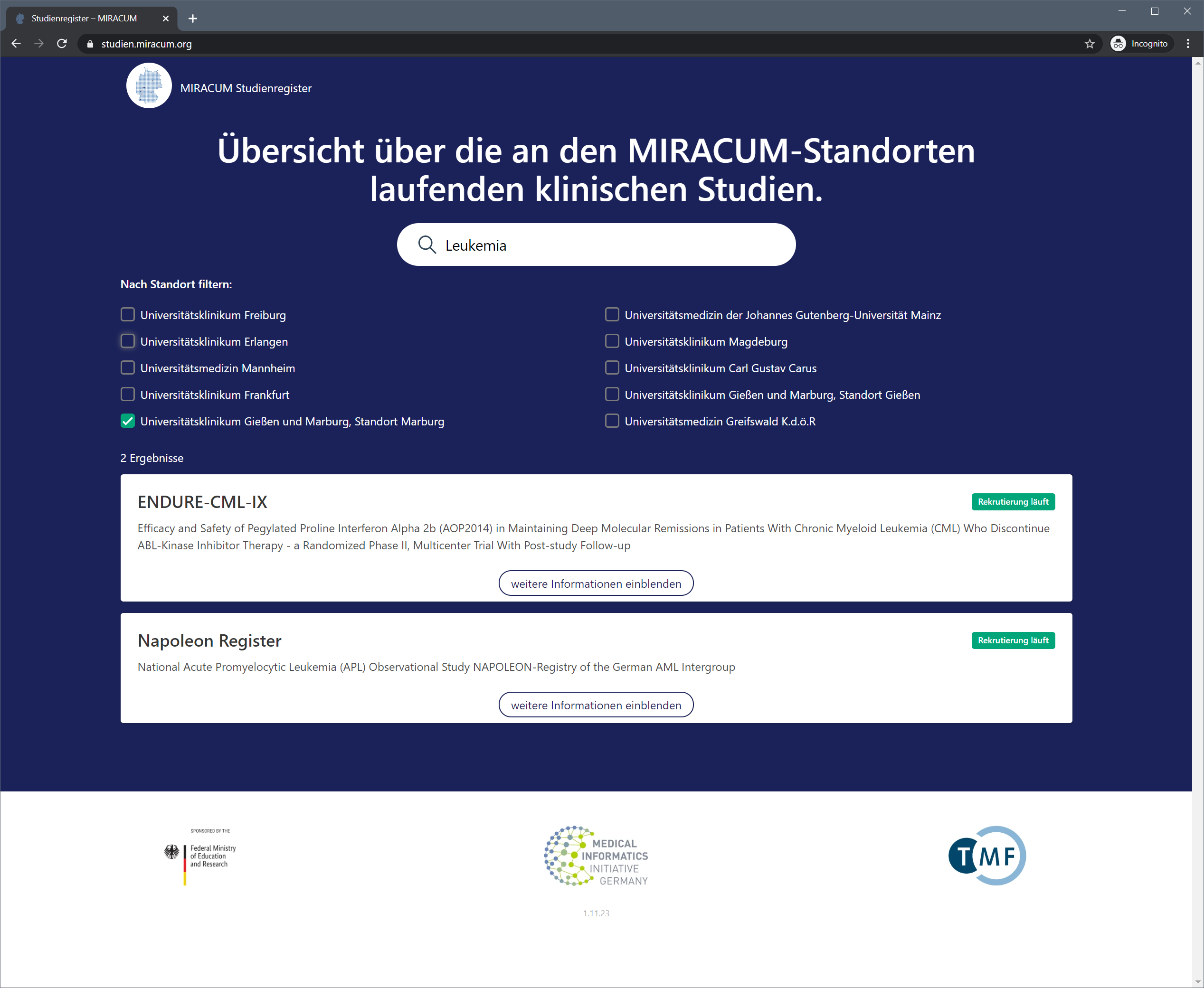

Supplement: Multimedia Appendix 2 [file medinform_v9i1e20470_app2.docx]
